# The Clinical Value of Pulmonary Rehabilitation in Reducing Postoperative Complications and Mortality of Lung Cancer Resection: A Systematic Review and Meta-Analysis

**DOI:** 10.3389/fsurg.2021.685485

**Published:** 2021-09-22

**Authors:** Xiaowei Mao, Yiqian Ni, Yanjie Niu, Liyan Jiang

**Affiliations:** Pulmonary and Critical Care Medicine, Shanghai Jiao Tong University, Shanghai Chest Hospital, Shanghai, China

**Keywords:** pulmonary rehabilitation, pulmonary resection, postoperative complications, mortality, meta-analysis

## Abstract

**Background:** Pulmonary rehabilitation is one meaningful way of improving exercise tolerance and pulmonary function. Thus, it may reduce the postoperative complications and mortality of pulmonary resection. Hence, we refreshed the data and conducted this systemic analysis.

**Method:** We searched Pubmed, Web of Science, and EMBASE using “lung OR pulmonary” AND “operation OR resection OR surgery” AND “rehabilitation or exercise.” The cut-off date was September 30, 2020. The publications were filtrated, and data were extracted from all selected studies by two reviewers. Review Manger 5.1 and the fixed or random regression model were used for calculating the pooled odds ratio (OR).

**Result:** Finally, 13 publications were enrolled in this study. Among them, five publications reported mortality, nine reported postoperative complications, and seven reported postoperative pulmonary complications. The pooled OR of mortality was 1.32 [95% confidence interval (CI): 0.54–3.23] for the pulmonary rehabilitation group, the pooled OR of postoperative complications was 0.62 (95% CI: 0.49–0.79) for the pulmonary rehabilitation group, and the pooled OR of postoperative pulmonary complications was 0.39 (95% CI: 0.27–0.56) for the pulmonary rehabilitation group. Subgroup analysis revealed the perioperative pulmonary rehabilitation was the most important part.

**Conclusion:** Pulmonary rehabilitation may not affect the mortality of pulmonary resection patients, however, it could decrease the number of postoperative complications, especially pulmonary complications. Perioperative pulmonary rehabilitation was the most important part of the program.

Lung cancer was the most leading cause of cancer-related deaths in China and even around the World (1, 2). Among all cases of lung cancer, 80% were non-small cell lung cancer (NSCLC) (3). Radical operation was a valuable way for early-stage NSCLC patients in multidisciplinary team (4). Usually, lung cancer patient characteristics include old age (5), having a history of smoking, and suffering from cardiovascular or respiratory comorbidities (6). These characteristics were also known as negative impactors in surgical tolerability, and they increase the perioperative risk (7). Under current surgical techniques and nursing skills, postoperative pulmonary complications (PPCs) occurred in 20–30% of patients (8). PPCs were regarded as the main causes of prolonged length of hospital stay, increased hospitalization cost, and poor life quality.

Pulmonary rehabilitation was a meaningful intervention in the management of chronic obstructive pulmonary disease or other chronic respiratory diseases (9). In 2015, “An Official American Thoracic Society/European Respiratory Society Policy Statement: Enhancing Implementation, Use, and Delivery of Respiratory rehabilitation” defined pulmonary rehabilitation as “comprehensive intervention based on a thorough patient assessment followed by patient-tailored therapies that include, but are not limited to, exercise training, education, and behavior change, designed to improve the physical and psychological condition of people with chronic respiratory disease and to promote the long-term adherence to health-enhancing behaviors” (10). So, a well-designed pulmonary rehabilitation program should include exercise training, pharmacotherapy, smoking cessation, nutritional support, behavior change, health education, etc. (11). The National Institute of Health and Clinical Excellence guidelines on lung cancer also emphasized the need for rehabilitation programs before and after surgery, stating that the outcomes should include mortality, pulmonary complications, pulmonary function, etc. (12). This topic was frequently studied. Several studies had reported the clinical value of pulmonary rehabilitation in shortening the length of hospital stay and improving exercise tolerance (13–15). At the same time, there had been other studies not showing positive effects of pulmonary rehabilitation program (16, 17). Also, some systemic analyses tried to answer the question of the clinical significance of pulmonary rehabilitation during the peri-operative period (18–22). However, some studies only included a randomized controlled trial (RCT) for future calculation (22). In addition, the newest one was published in 2019, and it only enrolled the publications before June 2017 (21). In the last few years, some new pulmonary rehabilitation clinical trials have been reported, including some non-RCT trials.

Thus, in this study, we aim to update the records and conduct this systemic analysis to explore the clinical value of pulmonary rehabilitation in decreasing postoperative complications and mortality of pulmonary resection.

## Methods

### Literature Search

We carried out a computerized search of published research studies in the Medline, Embase, and Web of Science databases and the Cochrane Library with the following: “lung OR pulmonary” AND “operation OR resection OR surgery” AND “rehabilitation or exercise.” Alternative spellings and abbreviations were also considered. Reference lists of included studies and relevant reviews were also manually searched. The literature search was conducted without any limitations. The publication date boundaries were January 1, 2005, and September 30, 2020.

All publications in English were considered. Conference abstracts or letters to editors were excluded due to their limited data. No minimum number of patients for a study was required to be included in our meta-analysis.

### Inclusion Criteria and Exclusion Criteria

All potentially relevant studies that met the following criteria were retrieved and assessed for inclusion: (1) the study should include the pulmonary rehabilitation and control group; (2) the outcome of the study should be one of the last items (postoperative complications, post-operative pulmonary complications, and mortality); (3) the study should include sufficient data for calculation. The exclusion criteria were as follows: (1) part of patients enrolled in the study not having received surgery.

If the same study cohort appeared in several articles, only the latest article was selected. Disagreements were resolved by discussion.

### Data Extraction

Data were extracted from all selected studies by two reviewers who worked independently, using a standardized form to ensure that all relevant information was captured. The following data were extracted from each publication: author, publication year, country, study design, pre- or post-operation, number of each group, the pulmonary rehabilitation program, the frequency of pulmonary rehabilitation, the time of pulmonary rehabilitation, the choice of operation, tumor stage of the patients enrolled, post-pulmonary operation complications, postoperative pulmonary complications, and mortality. If data of the items mentioned above were not reported in the study, the item was treated as “not reported.” Two reviewers assessed the Quality Rating Scheme for Studies (23). The third author assessed the data and resolved the disagreement.

### Statistical Analysis

All calculations were carried out with Review Manger 5.1 statistical software. All the analysis was conducted according to the standard methods recommended for a meta-analysis of. For each study, we calculated the odds ratio (OR) with 95% confidence interval (CI) to summarize the effects of pulmonary rehabilitation programs on postoperative morbidity and mortality. The fixed or random regression model was applied, and *P* < 0.05 was regarded as statistically significant. *I*^2^ statistics were used to detect statistically significant heterogeneity across the studies. Heterogeneity was evaluated by *I*^2^: if *I*^2^ > 50%, an article was considered to display substantial heterogeneity, requiring subgroup analysis. The potential publication bias was estimated by Deeks' funnel plots. A statistically significant publication bias existed if the *P*-value was <0.1 (24).

Begg's tests were used to detect any potential publication bias within the meta-analyses. The Begg's funnel plot showed the presence of bias visually.

## Results

### Study Selection

**Figure 1:**
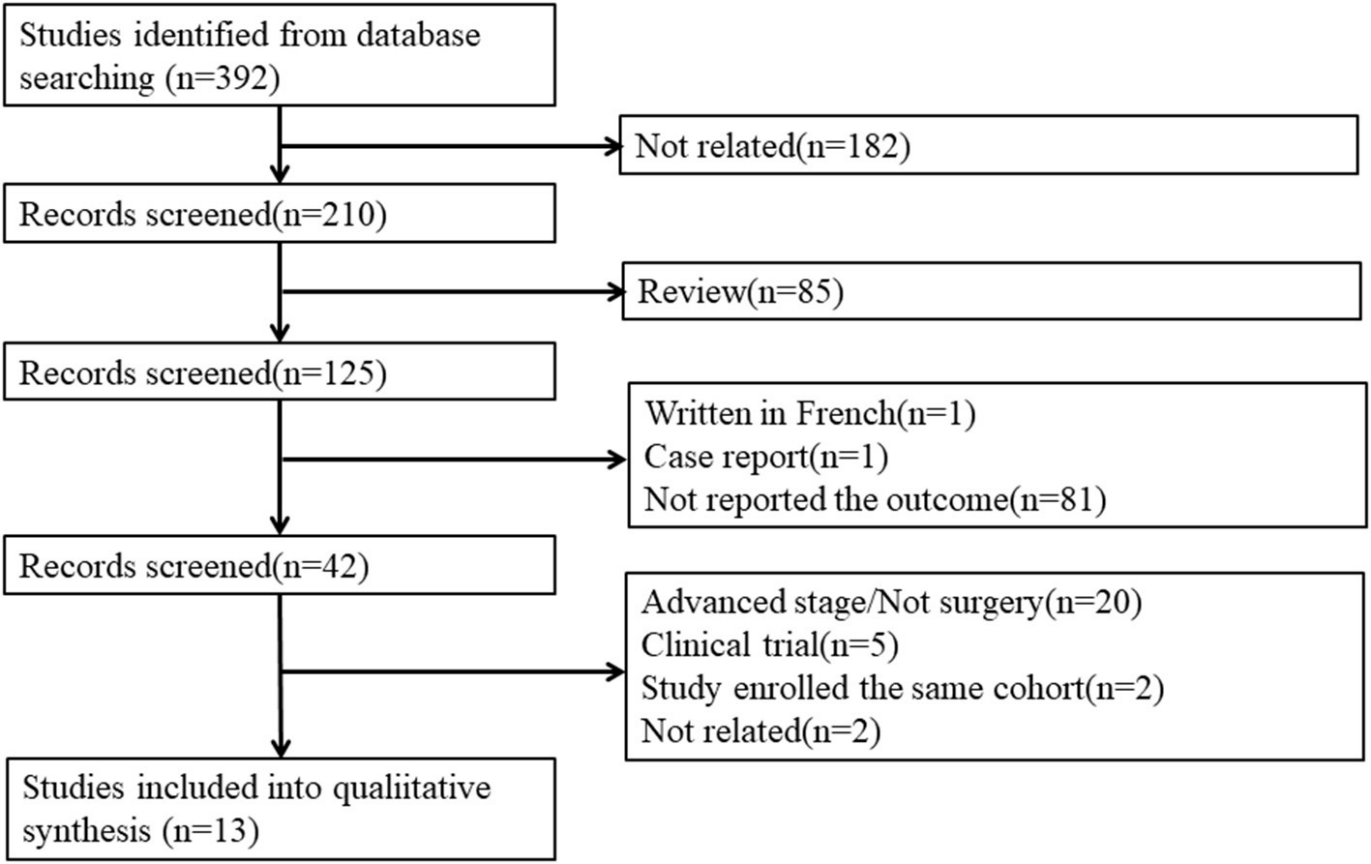
The flow chart of the publication selection.

Our search strategy identified 392 publications for consideration. Among these, 182 were irrelevant studies, and 85 reviews were removed. Then, the abstracts were reviewed: 81 studies were excluded because they did not report the three outcomes, 1 was written in French, and 1 was a case report. Of the 42 remaining publications, the full articles were obtained and reviewed, and another 29 studies were excluded for the following reasons: 20 studies were excluded because they enrolled advanced stage patients or not all patients received surgery, five studies were clinical trial protocol reports, two studies enrolled the same cohort, and two studies were not related to our study (Figure 1). Finally, 13 publications meeting all of the inclusion criteria were considered for the meta-analysis. Among them, five publications reported mortality, nine reported postoperative complications, and seven reported postoperative pulmonary complications.

### Study Descriptions and Quality Assessment

The 13 publications enrolled 2,501 patients totally. Among them, eight studies were prospective designs, three were retrospective, and the remaining two did not report. Six studies reported surgery method (video-assisted thoracoscopic surgery (VATS) or open), seven reported the surgery type (wedge resection, sleeve resection, segmentectomy, lobectomy, bilobectomy, and pneumonectomy), and six studies reported the cancer stage. In seven studies, the pulmonary rehabilitation was conducted before surgery, in two studies it was conducted after surgery, and in the remaining four studies, it was performed both pre- and post-operation. All the 13 studies adopted at least one exercise training, six studies adopted physiotherapy, and three studies adopted bronchodilators or antibiotics. Besides, healthy education was added to five studies, and nutritional intervention was used in one study. Smoking cessation was emphasized in five studies. The details were showed in Tables 1–3 and Supplement Table 1.

**Table 1:** Baseline characteristics.

**year**	**Author**	**Design**	**Group**	**Number**	**Sex**	**Age**	**Surgery method**		**Surgery type**			**Stage**					**References**
					**(Male)**	**(Male)**											
							**Open**	**VATS**	**WR/SR/ST**	**LB**	**BL/PN**	**I**	**II**	**III**	**IV**	**Unknown**	
2011	Roberto	Prospective	Rehabilitation	9													(25)
	Benzo		Control	8													
2011	Esra	Prospective	Rehabilitation	30		54.1			19	11	0						(26)
	Pehlivan		Control	30		54.76			24	6	0						
2011	Gill	Unknown	Rehabilitation	26		65.4			26			15	6	2		3	(27)
	Arbane		Control	25		62.6			25			10	6		5	4	
2013	Amy	Prospective	Rehabilitation	58	31	69											(28)
	Bradley		Control	305	182	67											
2014	G. Arbane	Prospective	Rehabilitation	67	32	67	45	19				24	12	6	7	15	(29)
			Control	68	44	68	45	19				29	12	8	2	16	
2015	Ke Gao	Prospective	Rehabilitation	71	40	66.33	29	42				26	39	4	2		(13)
			Control	71	44	59.67	32	39				41	19	11	0		
2015	Oliwia	Prospective	Rehabilitation	215	113	59^*^			61	7	147						(30)
	Glogowska		Control	187	102	55^*^			46	5	136						
2015	Natasa	Unknown	Rehabilitation	56	49	62			43	13							(14)
	Mujovic		Control	47	41	59			42	5							
2016	Gemma	Prospective	Rehabilitation	33		64.5											(31)
	CT		Control	9		75											
2016	Marc	Prospective	Rehabilitation	74	41	64		12	49	13	12	33	28	13			(32)
	Licker		Control	77	50	64		14	46	17	14	40	27	10			
2017	Zhou Kun	Retrospective	Rehabilitation	197	116	58.5	75	122	197	0	0	102	69	24	2		(33)
			Control	742	406	58.8	253	489	742	0	0	350	303	81	8		
2017	Hajime	Retrospective	Rehabilitation	31	27	72	20	11	31	0	0	18	8	5			(34)
	Saito		Control	31	27	71.3	18	13	31	0	0	20	6	5			
2018	Fairuz	Retrospective	Rehabilitation	19	15	65	4	15									(35)
	Boujibar		Control	15	10	69	2	13									

*VATS, video-assisted thoracoscopic surgery; WR, wedge resection; SR, sleeve resection; ST, segmentectomy; LB, lobectomy; BL, bilobectomy; PN, pneumonectomy*.

* *: median age*.

**Table 2:** Pulmonary rehabilitation program of each study.

**Year**	**Author**	**Pre/ post surgery**	**Time**	**Frequency**	**Physiotherapy**			**Bronchodilators/ antibiotic**		**Healthy education**	**Smoking cessation**	**Nutritional intervention**
					**Coughing exercise/ airway clearance**	**Inhalation therapy**	**Oxygen inhalation**					
2011	Roberto Benzo	Pre		Total 10 sessions								
2011	Esra Pehlivan	Pre	1 w		✓							
2011	Gill Arbane	Post			✓							
2013	Amy Bradley	Pre+post			✓	✓	✓			✓	✓	
2014	G. Arbane	Post										
2015	Ke Gao	Pre										
2015	Oliwia Glogowska	Pre+post			✓					✓		
2015	Natasa Mujovic	Pre+post	2–4 w	5/w	✓	✓		✓				
2016	Gemma CT	Pre						✓		✓	✓	
2016	Marc Licker	Pre									✓	✓
2017	Zhou Kun	Pre								✓		
2017	Hajime Saito	Pre+post	2–4 w	5/w	✓			✓			✓	
2018	Fairuz Boujibar	Pre	3–5 w							✓	✓	
**Year**	**Author**	**Exercise training**										**References**
		**Upper arm training**	**Lower arm training (including walking, treadmill)**	**Abdominal respiration/ diaphragmatic breathing**	**Pursed lip**	**Segmental breathing/ deep breathing**	**Thoracic cage expansion**	**Other IMT**	**Cycle ergometry**	**Incentive spirometry**	**Exercise -not otherwise**	
2011	Roberto Benzo	✓	✓					✓		✓		(25)
2011	Esra Pehlivan			✓	✓	✓				✓		(26)
2011	Gill Arbane										✓	(27)
2013	Amy Bradley					✓						(28)
2014	G. Arbane	✓							✓			(29)
2015	Ke Gao		✓							✓	✓	(13)
2015	Oliwia Glogowska	✓	✓			✓			✓		✓	(30)
2015	Natasa Mujovic			✓			✓				✓	(14)
2016	Gemma CT		✓					✓	✓			(31)
2016	Marc Licker		✓						✓		✓	(32)
2017	Zhou Kun			✓		✓					✓	(33)
2017	Hajime Saito			✓			✓	✓	✓		✓	(34)
2018	Fairuz Boujibar	✓	✓								✓	(35)

*IMT, Inspiratory Muscle Training*.

**Table 3:** Postoperative outcomes.

**Year**	**Author**	**Group**	**No. of patients**	**Mortality**		**PPCs**		**PPCs of lung**		**References**
				**No**.	***p***	**No**.	***p***	**No**.	***p***	
2011	Roberto Benzo	Rehabilitation	9					3	0.23	(25)
		Control	8					5		
2011	Esra Pehlivan	Rehabilitation	30			1	0.04			(26)
		Control	30			5				
2011	Gill Arbane	Rehabilitation	26			2	NS			(27)
		Control	25			3				
2013	Amy Bradley	Rehabilitation	58	2	0.62			5	0.21	(28)
		Control	305	6				49		
2014	G. Arbane	Rehabilitation	67			20		10		(29)
		Control	68			22		16		
2015	Ke Gao	Rehabilitation	71			12	0	5	0.0004	(13)
		Control	71			59		25		
2015	Oliwia Glogowska	Rehabilitation	215			32	0.19			(30)
		Control	187			37				
2015	Natasa Mujovic	Rehabilitation	56	2	0.191	20	0.354	17	0.2	(14)
		Control	47	0		21		20		
2016	Gemma CT	Rehabilitation	33	0	0.05					(31)
		Control	9	1						
2016	Marc Licker	Rehabilitation	74	2	0.64	27	0.08	17	0.01	(32)
		Control	77	2		39		33		
2017	Zhou Kun	Rehabilitation	197	2	0.611	36	0.022			(33, 36)
		Control	742	4		194				
2017	Hajime Saito	Rehabilitation	31			2		2		(34)
		Control	31			5		5		
2018	Fairuz Boujibar	Rehabilitation	19			8	0.038			(35)
		Control	15			12				

*PPCs, post-pulmonary operation complications*.

The data of quality assessment was showed in Supplementary Figure 1.

### Mata-Analysis and Systemic Review

For postoperative complications analysis, nine studies enrolled 1,937 patients. The pooled OR was 0.62 (95% CI: 0.49–0.79), favoring the pulmonary rehabilitation. For subgroup analysis, we found the pre-surgery rehabilitation had a clinical significance, and the post- or pre-+post- only showed tendencies in favor of pulmonary rehabilitation.

For postoperative pulmonary complications analysis, seven studies enrolled 969 patients. The pooled OR was 0.39 (95% CI: 0.27–0.56) favoring the pulmonary rehabilitation. For subgroup analysis, we found the pre-surgery and pre-+post-surgery rehabilitation subgroups had a clinical significance, and the post-surgery subgroup only showed a tendency in favor of pulmonary rehabilitation.

For mortality analysis, five studies enrolled 1,598 patients. The pooled OR was 1.32 (95% CI: 0.54–3.23), no clinical significance was showed in rehabilitation group. For subgroup analysis, both the pre-surgery and pre-+post-surgery rehabilitation subgroups showed no difference in rehabilitation or control group.

**Figure 2:**
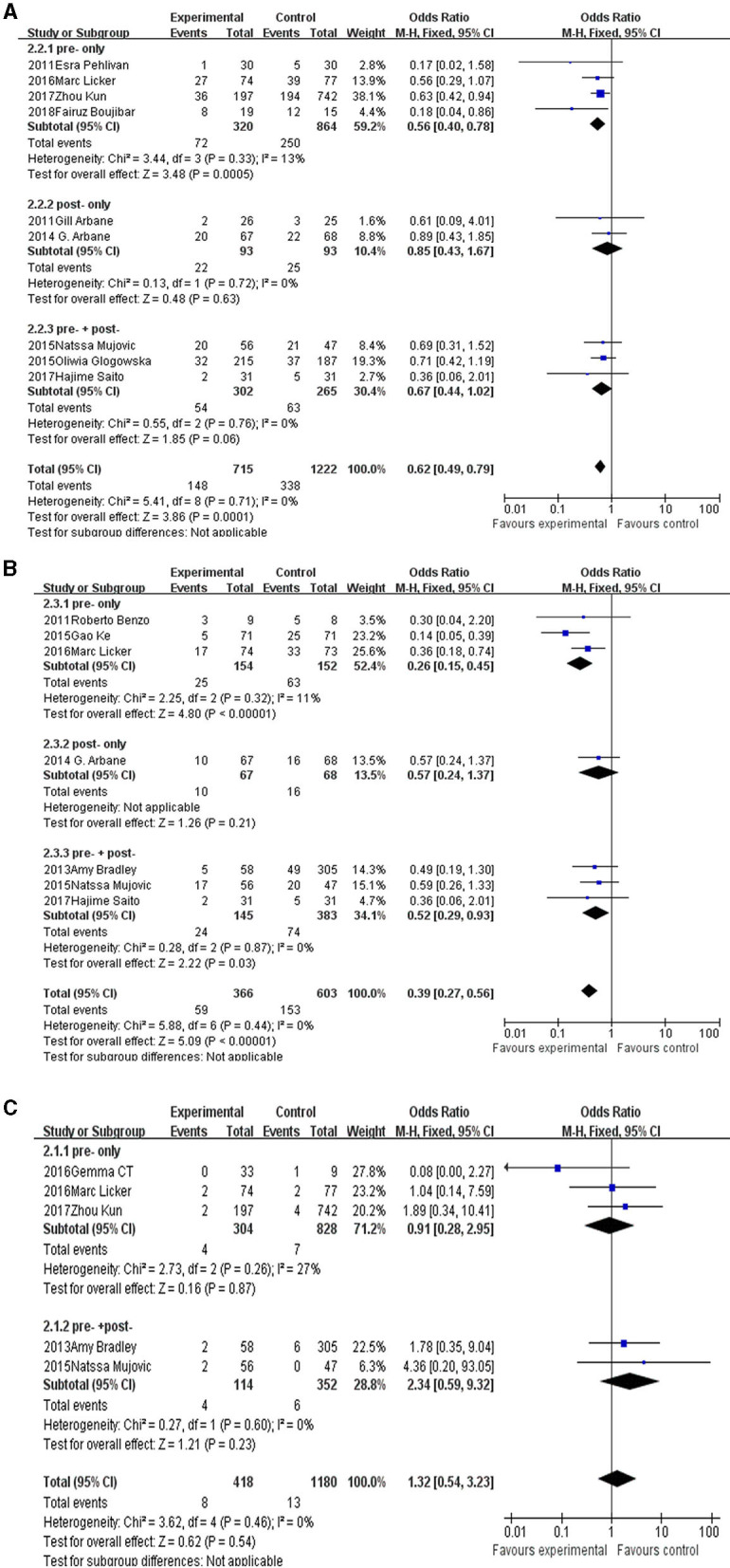
The forest plot of postoperative complications **(A)**, postoperative pulmonary complications **(B)**, and mortality **(C)**.

The details were showed in Figures 2A–C.

All three analyses showed no publication bias. The *I*^2^ < 50% and *P* > 0.01 in those three analyses. The funnel plot was showed in Supplementary Figures 2–4.

## Discussion

Surgery operation remains the optimal selection for early-stage lung cancer patients, and it was also a crucial part of a multidisciplinary team for advanced lung cancer patients. Lung cancer was related to smoking history, thus the patients always had chronic lung disease, heart disease, and cerebrovascular diseases at the same time (6, 36). Those risk factors may increase the PPCs after pulmonary operation (7). Besides, lung cancer patients suffered deconditioning, muscle weakness, fatigue, cachexia, and anxiety, those sufferings resulted in disability and impaired quality of life among lung cancer individuals (37, 38). Pulmonary rehabilitation was usually applied in chronic obstructive pulmonary disease, and it was significantly associated with a lower risk of death (39, 40). Pulmonary rehabilitation was also recommended for other chronic pulmonary diseases, interstitial lung disease, cystic fibrosis, lung cancer, etc. (10, 11). Several studies have supported the positive effects of rehabilitation in muscle strength, exercise endurance, well-being, and health status (25, 41–43), and it also relieved the discomfort from symptoms (44, 45). In recent years, pulmonary rehabilitation had been advocated by a wide range of surgical specialties, including cardiothoracic surgery. Many single-center-based studies have reported the clinical values of pulmonary rehabilitation. For those who would undergo pulmonary operations, the pulmonary rehabilitation program could apply before surgery, after surgery, or both pre- and post- surgery. For preoperative pulmonary rehabilitation, it can improve individuals' exercise tolerance and overall medical stability before surgery resection (46, 47). Those who received pulmonary rehabilitation after lung cancer resection surgery may gain increasements in walking endurance, peak exercise capacity, and decrease in dyspnea and fatigue (48, 49). At some centers, the pulmonary rehabilitation was applied during hospitalization (14, 28, 30).

Many studies had supported the positive roles of rehabilitation in decreasing postoperative complications and mortality, but the majority of them are based on a single center and a limited number of patients, and they thus could not avoid selection bias. The latest systemic analysis was published in 2019 and only enrolled publications before June 2017 (21). We therefore conducted this study to update the records and explore the clinical value of pulmonary rehabilitation in decreasing postoperative complications and mortality.

After selection, nine studies enrolled 1,937 patients in total and reported postoperative complications, and seven studies enrolled 969 patients in total and reported pulmonary complications. In our study, pulmonary rehabilitation had proved the clinical values in decreasing the postoperative complications for patients, especially pulmonary complications. Previous research suggested that pulmonary function was a good predictor for pulmonary resection, including, for example, the forced expiratory volume in one second (FEV1), forced vital capacity (FVC), carbon monoxide diffusing capacity (DLCO), the cardiopulmonary exercise test (CPET), impulse oscillometry (IOS), etc. (50–54). Pulmonary rehabilitation has been proven to improve the cardiopulmonary function, exercise tolerance, anxiety, depression, etc. (46, 55–61). Lai et al. suggested that pre-surgery pulmonary rehabilitation may improve the FEV1, FVC, and 6-minute walking test (6MWT) (58, 59). Jones's study, apart from pulmonary function, observed an improvement in cardiopulmonary function after presurgical exercise training (46). Stefanelli et al. measured using the BROG scale and found the modified breath in chronic obstructive pulmonary disease (COPD) patients after high-intensity training and cardiopulmonary exercise. In Cavalheri's study, post-surgery pulmonary rehabilitation showed positive values in pulmonary function, cardiopulmonary function, and mental fitness (57). Vagvolgyi's study also demonstrated the clinical value of post-surgery pulmonary rehabilitation (60). Besides, pulmonary rehabilitation may decrease the level of cytokine and inflammation factors. In Messaggi-Sartor's study, after an 8-week training program, an increase of 0.61 μg/mL in the serum IGFBP-3 levels for patients in the intervention group was observed (61). Fiorelli et al. reported a lower level of Serum IL-6 (*P* = 0.001), IL-10 (*P* = 0.001), and TNF-α (*P* = 0.001) in the transcutaneous electrical nerve stimulation group than in the control group (55). In our analyses, we showed a positive result of pulmonary rehabilitation, especially in the pre-surgery subgroup. This may be because the outcome of this study was the main complications after surgery. Pre-surgery rehabilitation improved pulmonary function and cardiopulmonary function before an operation, thus decreasing complications after surgery. For the post-operation rehabilitation subgroup, it showed a favoring of the pulmonary rehabilitation group, but the result was not statistically significant. We inferred that the complications occurred before the pulmonary rehabilitation worked. We suggested the pre-surgery pulmonary rehabilitation should be operated as perioperative interventions, especially for high-risk patients. The main goal of perioperative rehabilitation is to improve pulmonary function, avoiding atelectasia, pneumonia, etc. Herin, apart from calculating the pooled effect of the postoperative complications, we specifically calculated the pooled OR of decreasing postoperative pulmonary complications. We found the pulmonary rehabilitation worked better in decreased PPCs than total complications. This may be because the rehabilitation program focuses on the lung.

Some studies have argued for the positive clinical value of pulmonary rehabilitation in long-term survival for those pulmonary resection patients (61, 62). While perioperative rehabilitation would improve lung function, other organs would gain beneficence from this procedure, such as the heart. Mortality related to heart disease and related issues would decrease. But in this study, the pulmonary rehabilitation did not show the clinical value for mortality in those who received pulmonary surgery in either the pre-operative group or pre- +post- group. As mentioned above, pulmonary rehabilitation could improve cardiopulmonary function, exercise tolerance, etc. Those factors also were effective predicted factors for mortality, such as DLCO (63, 64). Both pre- and post-surgery pulmonary rehabilitation showed an improvement in DLCO (56, 65). In our study, no significant value of pulmonary rehabilitation in reducing mortality was observed, several reasons may account for the result. Firstly, only five studies were enrolled in this meta-analysis, limited people were enrolled, especially the rehabilitation group. Secondly, it could be attributed to the development of surgical techniques. Among them, two studies reported on surgical methods. In Licker's study, all patients received VATS. In Zhou's study, more than half of the patients performed VATS. This means low mortality would be observed in those cohorts. Thirdly, some studies were not RCT, so select bias could not be avoided.

Our study also had some limits. Firstly, for defined outcomes, only a few studies were included in the meta-analysis. This may result in publication bias. Secondly, some studies were not randomized controlled trials, and this may cause selected bias when conducted the clinical trial. Thirdly, the studies enrolled were mostly performed in one center, which also resulted in select bias. Forth, it is difficult to divide complications directly related to surgery from those related to comorbidity, and we summarize the complications as PPCs and total complications.

Summarily, pulmonary rehabilitation is meaningful in avoiding postoperative complications of pulmonary resection. We suggested that pulmonary rehabilitation should be included in the perioperative period, and perioperative pulmonary rehabilitation was the most important part of the program. Also, a more well-designed RCT is required to provide proof of our results.

## Data Availability Statement

The original contributions generated for the study are included in the article/Supplementary Materials, further inquiries can be directed to the corresponding author.

## Author Contributions

XM, YNi, and YNiu conducted the literature search and data extraction. XM wrote the manuscript. YNi revised the manuscript. LJ reviewed the manuscript and directed and supervised the study. All authors contributed to the article and approved the submitted version.

## Funding

This work was supported by the National Key Research and Development Program of China: 2018YFC1313600 and the Chinese Society of Clinical Oncology (CSCO): Y-2019AZZD-0038.

## Conflict of Interest

The authors declare that the research was conducted in the absence of any commercial or financial relationships that could be construed as a potential conflict of interest.

## Publisher's Note

All claims expressed in this article are solely those of the authors and do not necessarily represent those of their affiliated organizations, or those of the publisher, the editors and the reviewers. Any product that may be evaluated in this article, or claim that may be made by its manufacturer, is not guaranteed or endorsed by the publisher.

## Supplementary Material

Supplement Table 1 Main pulmonary rehabilitation protocols.

Supplementary Figure 1 Quality rating scheme for enrolled studies.

Supplementary Figure 2 Funnel plot of postoperative complications.

Supplementary Figure 3 Funnel plot of postoperative pulmonary complications.

Supplementary Figure 4 Funnel plot of mortality.
