# Supplementary material for: The Clinical Value of Pulmonary Rehabilitation in Reducing Postoperative Complications and Mortality of Lung Cancer Resection: A Systematic Review and Meta-Analysis
Source: Front Surg. 2021 Sep 22;8:685485. doi: 10.3389/fsurg.2021.685485 (PMC8503917; doi:10.3389/fsurg.2021.685485)
Supplement: Supplementary file 1 [file Data_Sheet_1.doc]

**PRISMA-P (Preferred Reporting Items for Systematic review and Meta-Analysis Protocols) 2015 checklist: recommended items to address in a systematic review protocol***

| Section and topic | Item No | Checklist item |
| --- | --- | --- |
| ADMINISTRATIVE INFORMATION | | |
| Title: |  |  |
| Identification | 1a | Yes. |
| Update | 1b | Yes. |
| Registration | 2 | No. |
| Authors: |  |  |
| Contact | 3a | Yes. |
| Contributions | 3b | Yes. |
| Amendments | 4 | No. |
| Support: |  |  |
| Sources | 5a | Yes. |
| Sponsor | 5b | Yes. |
| Role of sponsor or funder | 5c | They didn’t attend this study. |
| INTRODUCTION | | |
| Rationale | 6 | Yes. |
| Objectives | 7 | Yes. |
| METHODS | | |
| Eligibility criteria | 8 | Yes. |
| Information sources | 9 | Yes. |
| Search strategy | 10 | Yes. |
| Study records: |  |  |
| Data management | 11a | Yes. |
| Selection process | 11b | Yes. |
| Data collection process | 11c | Yes. |
| Data items | 12 | Yes. |
| Outcomes and prioritization | 13 | Yes. |
| Risk of bias in individual studies | 14 | Yes. |
| Data synthesis | 15a | Yes. |
|  | 15b | Yes. |
|  | 15c | Yes. |
|  | 15d | Yes. |
| Meta-bias(es) | 16 | Yes. |
| Confidence in cumulative evidence | 17 | Yes. |
