# Supplementary material for: The Clinical Value of Pulmonary Rehabilitation in Reducing Postoperative Complications and Mortality of Lung Cancer Resection: A Systematic Review and Meta-Analysis
Source: Front Surg. 2021 Sep 22;8:685485. doi: 10.3389/fsurg.2021.685485 (PMC8503917; doi:10.3389/fsurg.2021.685485)
Supplement: Supplement Table 1 — Main pulmonary rehabilitation protocols. [file Data_Sheet_2.docx]

Supplement Table1: Main pulmonary rehabilitation protocols

| year | Author | number | Ref. |
| --- | --- | --- | --- |
|  |  |  |  |
| 2011 | Roberto Benzo | Lower extremity (LE) endurance training, Upper extremity (UE), Strengthening exercises with Thera-band, Inspiratory muscle training (IMT), slow breathing endurance. | 26 |
| 2011 | Esra Pehlivan | Chest physiotherapy (diaphragmatic, pursed lip, segmental breathing exercise, usage of incentive spirometry, coughing exercise) and walking exercise, Routine physical therapy. | 27 |
| 2011 | Gill Arbane | Usual care including pain relief, strength and mobility (walking, as able, marching on the spot and recumbent bike exercises) training. | 28 |
| 2013 | Amy Bradley | Education and self-management, endurance and strength exercises as well as inspiratory muscle exercises, Smoking cessation, nutritional intervention, sitting out of bed, early mobilization, deep breathing exercises, assisted coughing augmented by nebulizers and humidified oxygen. | 29 |
| 2014 | G. Arbane | Standard care, cycle and strength training, home walking program. | 30 |
| 2015 | Ke Gao | Drug intervention (antibiotic, bronchodilator, corticosteroid, expectorant treatment ), Respiratory physiological adaptability training(abdominal breath, Breath training device, lower extremity endurance training ). | 31 |
| 2015 | Oliwia Glogowska | Education, deep breathing exercises, vibration massage, coughing exercises, Upper / Lower -limb exercise training, Controlled breathing training, marching in place, anti-edematous prevention, Walking down the hall, Training on the bike / stairs / Stairmaster. | 32 |
| 2015 | Natasa Mujovic | Intravenous bronchodilators, pulmonary physiotherapy (Salbutamol aerosols) and general physiotherapy, thoracic cage expansion and shoulder mobilization, diaphragmatic (abdominal) breathing. | 33 |
| 2016 | Gemma CT | Respiratory muscle training and breathing exercises (strengthening the muscles of respiration; improving their diaphragmatic breathing and control; coordinating their breathing process; and retraining their respiratory muscles with the use of incentive spirometry), cardiovascular exercises (stationary cycle ergometry  and walking exercise training), education, bronchodilator therapy. | 34 |
| 2016 | Marc Licker | Cycling ergometer, additional exercises (leg press, leg extension, back extension, seat row, biceps curls, or chest and shoulder press). | 35 |
| 2017 | Zhou Kun | IMT exercise (abdominal breathing training, inspiration exercise), Aerobic endurance training (Nu-Step). | 36 |
| 2017 | Hajime Saito | Breathing and coughing techniques, diaphragmatic breathing, incentive respiratory exercise, peripheral muscle exercise training (cycle ergometer), peripheral circulation exercises, aerosol therapy with bronchodilators, chest expansion, shoulder girdle mobilization. | 37 |
| 2018 | Fairuz Boujibar | exercise retraining, lower and upper limbs muscular strengthening, therapeutic education, smoking cessation. | 38 |
