# Supplementary figures and images for: The Clinical Value of Pulmonary Rehabilitation in Reducing Postoperative Complications and Mortality of Lung Cancer Resection: A Systematic Review and Meta-Analysis
Source: Front Surg. 2021 Sep 22;8:685485. doi: 10.3389/fsurg.2021.685485 (PMC8503917; doi:10.3389/fsurg.2021.685485)

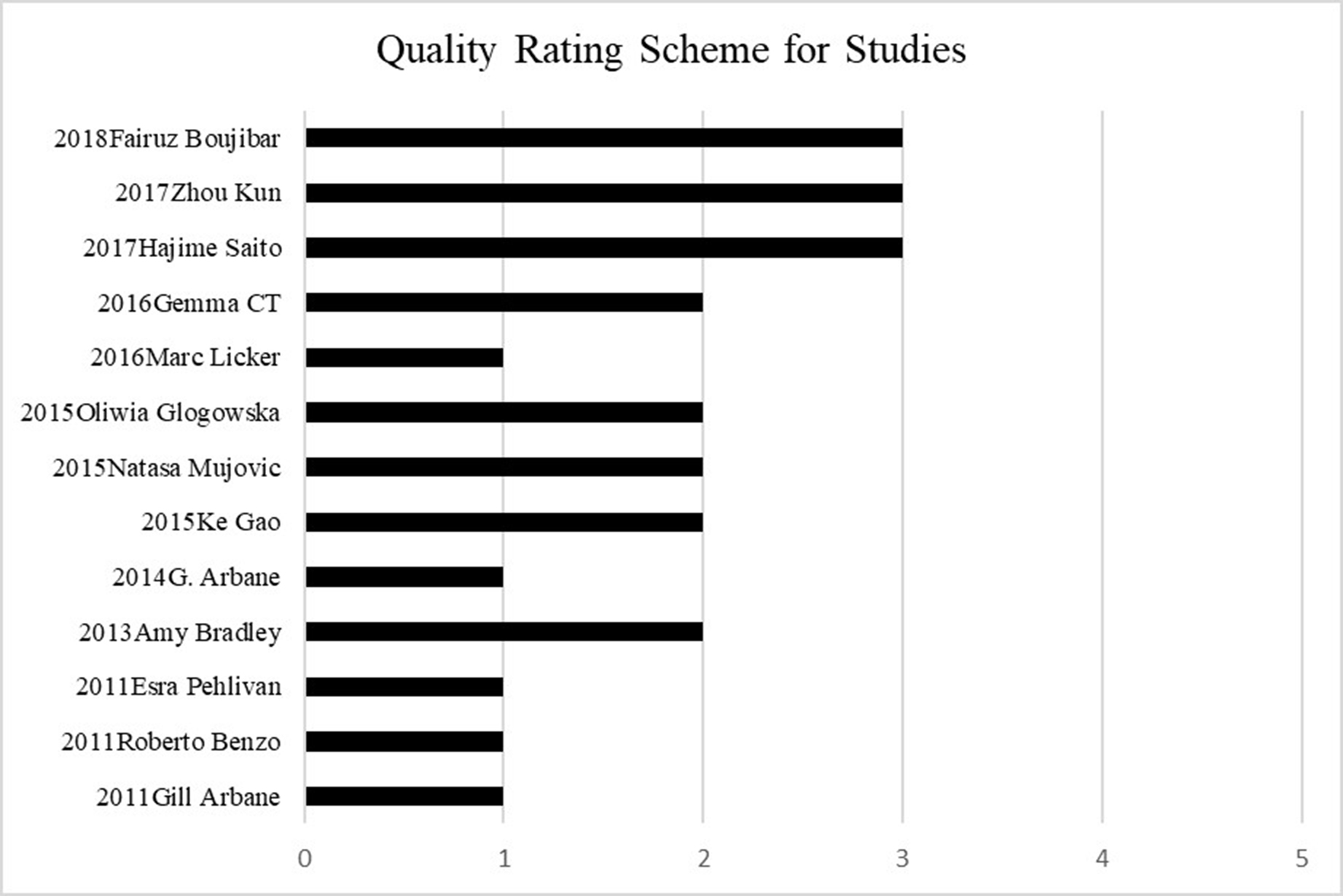

Supplement: Supplementary Figure 1 — Quality rating scheme for enrolled studies. [file Image_1.JPEG]

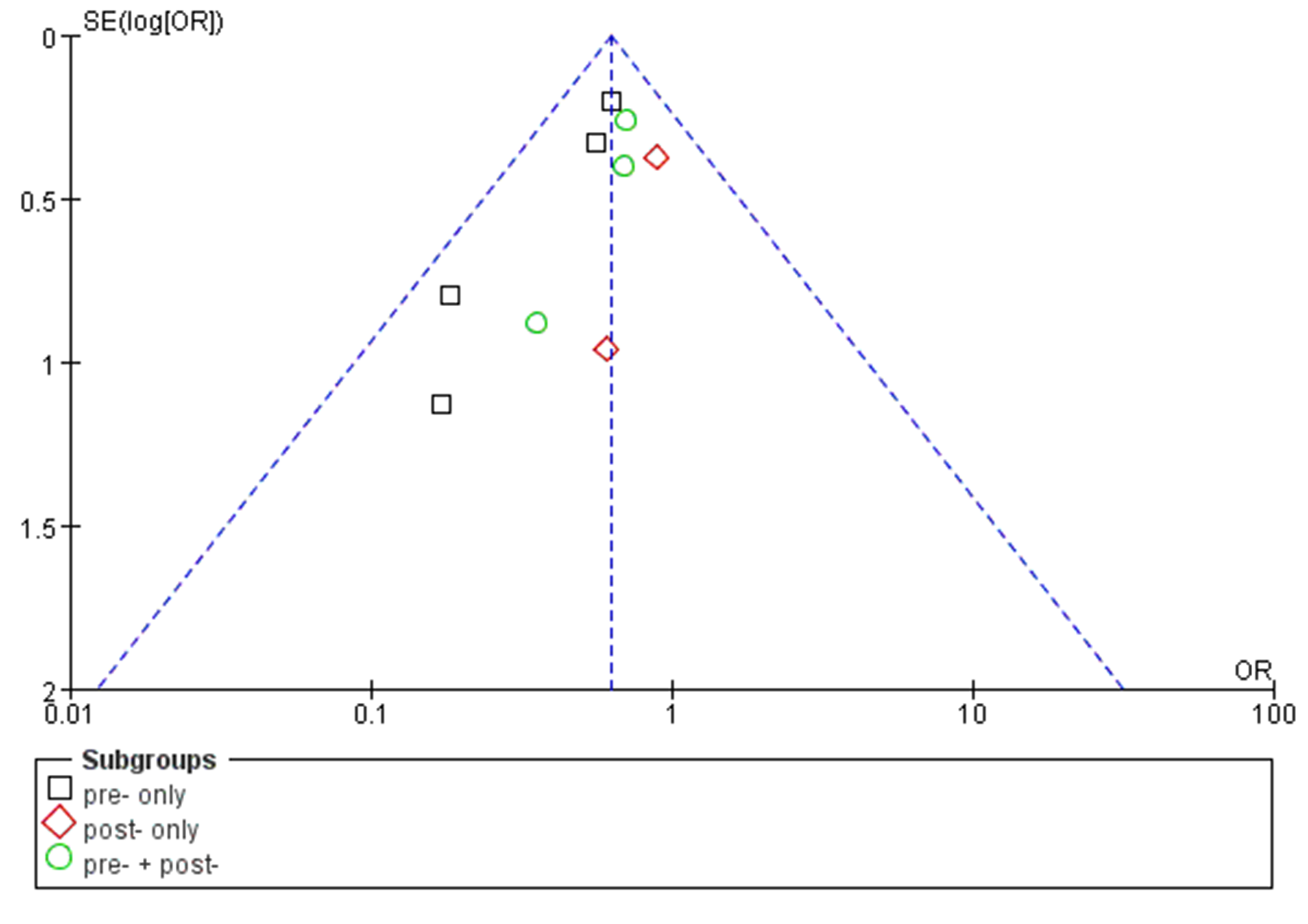

Supplement: Supplementary Figure 2 — Funnel plot of postoperative complications. [file Image_2.tiff]

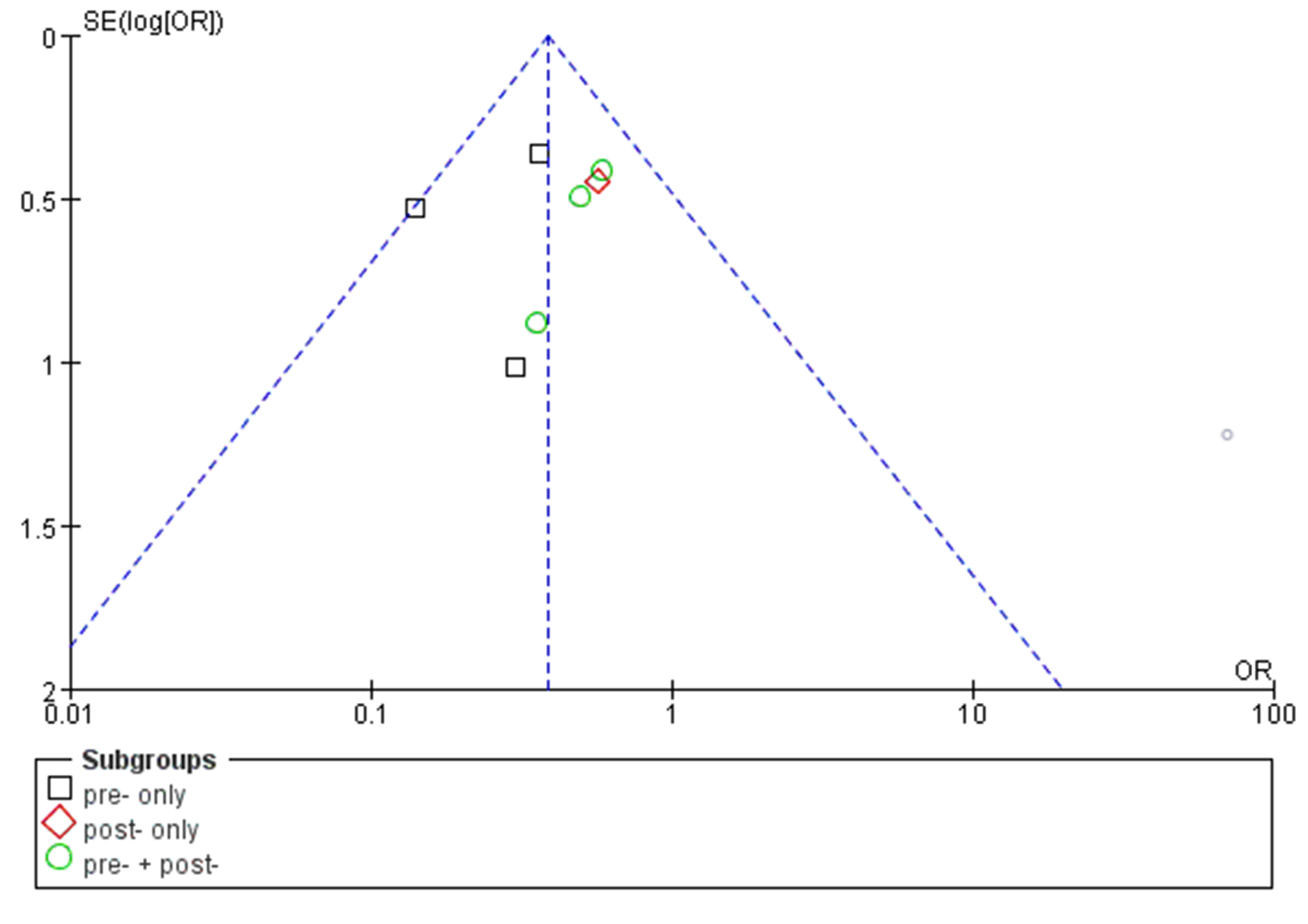

Supplement: Supplementary Figure 3 — Funnel plot of postoperative pulmonary complications. [file Image_3.tiff]

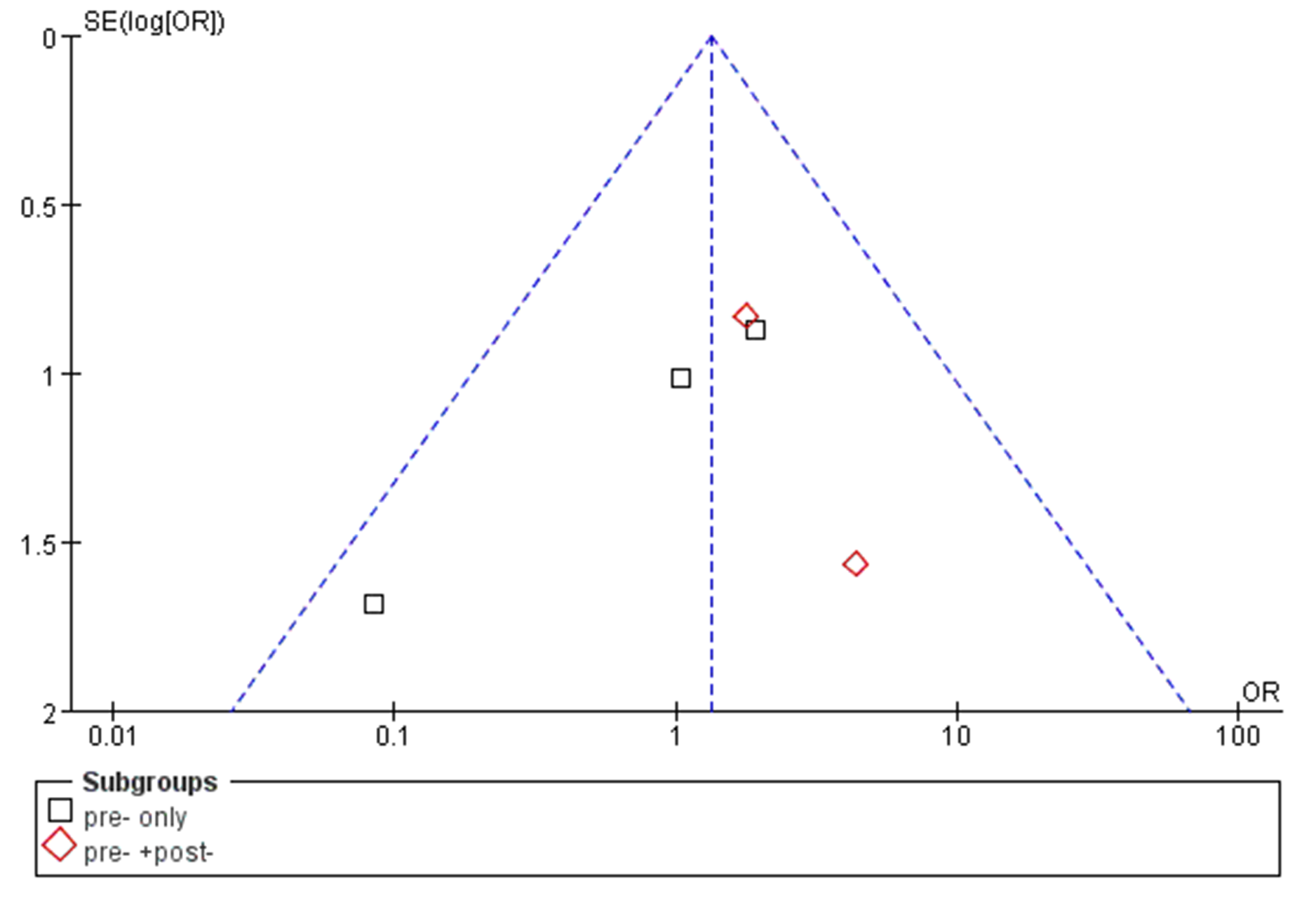

Supplement: Supplementary Figure 4 — Funnel plot of mortality. [file Image_4.tiff]
